# Afraid but misinformed: Conspiracist beliefs cancel the positive influence of fear of COVID-19 on vaccination intentions - Findings from a Romanian sample

**DOI:** 10.3389/fpsyg.2023.1109064

**Published:** 2023-04-20

**Authors:** Andrei C. Holman, Simona A. Popușoi

**Affiliations:** Department of Psychology, Faculty of Psychology and Education Sciences, Alexandru Ioan Cuza University of Iași, Iași, Romania

**Keywords:** COVID-19 risks, fear of COVID-19, conspiracist beliefs, vaccination intentions, ambivalence

## Abstract

Understanding the factors that make people more likely to refuse vaccination against COVID-19 is crucial in order to design public health messages efficient in increasing vaccination rates. As COVID-19 creates risks of seriously damaging health effects, fear of this disease is as a significant determinant of vaccination intentions, as indicated by past research. Nevertheless, this positive influence may be limited in people who do not consider vaccines as a solution to protect against COVID-19, especially those who hold conspiracist beliefs about the new coronavirus and, implicitly, about the newly developed vaccines. The present study examined in a cross-sectional design on a convenience sample (*N* = 564) the joint effect of fear of COVID-19 and conspiracist beliefs on vaccination intentions, advancing past research on their independent influences. Furthermore, we investigated and controlled the effects of perceived risk of catching COVID-19, trust in medical experts, attitude towards vaccination and socio-demographical characteristics (i.e., gender, age, and education), previously found to be associated to COVID-19 vaccination intentions. We also tested the effect of ambivalence towards vaccination, i.e., the degree to which people simultaneously hold positive and negative evaluations of this intervention, as the widespread misinformation on the new coronavirus and its vaccines may induce ambivalence on this latter issue in many people. The results showed that the positive effect of fear of COVID-19 on vaccination intentions emerged only in participants who tend not to endorse conspiracist ideas on the new coronavirus. Moreover, higher vaccine hesitancy was found in participants with higher ambivalence towards vaccination, in those who perceive the risk of being contaminated by the new coronavirus as low, and in those with more negative attitudes towards vaccines in general. Vaccine ambivalence also emerged as a mediator of the negative effects of conspiracist beliefs about COVID-19 on vaccination intentions. This pattern of findings suggests the public messages emphasizing the risks of COVID-19 should also combat misinformation in order to maximize vaccine uptake.

## 1. Introduction

Vaccine hesitancy has represented an important obstacle in the current global efforts to fight against the COVID-19 pandemic. Nevertheless, despite the benefit of vaccination of reducing public health risks, there have been major differences between countries in what regards COVID-19 vaccination rates, even within regions where vaccines are available and free for all the population, such as Europe. During the COVID-19 pandemic, Romania has ranked at the bottom of the COVID-19 vaccination rate in the European Union (EU). Currently (i.e., October 2022), 42.4% of the Romanian population has taken the complete primary course of vaccination against COVID-19 (i.e., two primary doses), far below the European Union (EU) mean of 75% (ECDC, 2022). Vaccine hesitancy has been highlighted as a significant problem for public health in the developed world before the current pandemic, as it was associated to the outbreak of several vaccine-preventable diseases (Salmon et al., 2015). Moreover, confidence in vaccines in general has been found to be lower in Europe in comparison to other continents (De Figueiredo et al., 2020). As such, past research has sought to identify the determinants of vaccine hesitancy, in order to inform interventions aiming to improve people’s attitudes and intentions towards vaccines, and ultimately to increase vaccination rates. During the current pandemic, many of these previously highlighted factors have been reexamined in relationship to people’s hesitancy to uptake the available COVID-19 vaccines.

Generally, the extant body of research indicates a wide range of social and individual factors of COVID-19 vaccine hesitancy, which includes perceived personal risks of this disease and the related anxiety, conspiracist beliefs about COVID-19, attitudes towards vaccines, trust in the relevant authorities, gender or education (e.g., Freeman et al., 2020; Allington et al., 2021; Bendau et al., 2021; Latkin et al., 2021; McNeil and Purdon, 2022; Sekizawa et al., 2022). While the effects of these factors on COVID-19 vaccine hesitancy have been examined independently in past research, the present study aims to investigate the intertwined influence of two important predictors in this set, as highlighted by previous results, namely COVID-19 fear and beliefs in the conspiracy theories about the new coronavirus. This may contribute to the understanding of the reasons why many people, even when confronting and being fearful about a potentially deadly or debilitating health risk, still refuse the readily available intervention that would offer them direct protection against it.

COVID-19 creates risks of adverse health outcomes and even death. Consequently, anxiety about the disease, instilled by the apprehension of these risks, can be conceived during the current pandemic as a “functional fear” that would presumably motivate compliance with public health regulations and acceptance of the medical interventions that significantly reduce the risk of this illness (i.e., vaccination; Harper et al., 2020). Past results support this idea, by highlighting positive associations between COVID-19 risk perception and anxiety, on the one hand, and vaccine acceptance on the other, in several countries, such as Germany (Bendau et al., 2021), Turkey, United Kingdom (Salali and Uysal, 2020), or France (Detoc et al., 2020). Similarly, intention to uptake COVID-19 vaccination was found to be higher in people who feel more intense fear in relation to this disease (Chu and Liu, 2021; Sekizawa et al., 2022) and in those who perceive it as a more severe health condition (Ruiz and Bell, 2021) or as more life threatening (Dror et al., 2020). Currently there is no published longitudinal research that would indicate changes in people’s fear of this disease. Moreover, as a recent review concludes, most of the studies including assessments of COVID-19-related fear were conducted during the initial phases of the pandemic, i.e., until May 2020 (Quadros et al., 2021). Nevertheless, we can presume that the increasing incidence and mortality worldwide has intensified to some extent the perceived risk of the new coronavirus and its associated emotional reaction of fear. Yet, studies that compared COVID-19 vaccination intentions across time since the outbreak of the pandemic indicate that the cumulative increase in COVID-19 caseloads did not produce an increase of people’s willingness to uptake the available vaccines (Al-Amer et al., 2022). Moreover, as noted above, there are countries in which a large share of the population still refuses the available COVID-19 vaccines. This suggests that in the specific context of the current pandemic, fear might have a limited effect on vaccination intentions in some individuals, which highlights the importance of revealing the factors that interfere with the health-protective influence of this functional fear. The present study examines conspiracist beliefs about COVID-19 as such a potentially interfering factor, in the theoretical framework of the Protection Motivation Theory (PMT, Rogers, 1975, 1983).

The PMT describes two types of appraisals as fundamental factors of individual’s motivation to engage in protective behaviors against a threat, such as vaccination against COVID-19. The first, threat appraisal, is mainly a result of one’s evaluation of the severity of the threat and of one’s vulnerability to the threat. People who perceive the threat as severe and who also believe that their chances of being affected are high are more motivated to adopt protective behaviors. Also, fear is strongly related to these appraisals, as perceiving oneself being exposed to a severe threat is associated to experiencing an intense fear (Rogers, 1975; Witte and Allen, 2000). The second type of appraisal concerns the individual’s coping appraisal, which further depends on perceived response efficacy (i.e., one’s evaluation of the efficiency of the recommended behavior in averting the threat), self-efficacy (i.e., one’s beliefs in one own’s ability to actually perform this behavior), and the perceived costs of the recommended course of action. People are more inclined to adopt the preventive behavior when they perceive it as efficient, they trust their ability to perform it and they evaluate it as incurring low effort, energy, financial and/or time costs. The PMT framework has been used for understanding people’s reactions to messages from public health authorities recommending actions deemed as protective against various health threats (i.e., *fear appeals*), such as seasonal influenza (e.g., Ling et al., 2019), SARS (e.g., Jiang et al., 2009) or cancer (e.g., Babazadeh et al., 2016), including the adoption of behaviors preventing contamination during the COVID-19 pandemic (Ezati et al., 2021; Kim et al., 2021).

As reviewed above, fear of COVID-19 has been found to make people more prone to uptake the vaccine against the new coronavirus, suggesting that individuals who perceive this disease as highly threatening experience more intense fear and are more inclined to adopt the recommended protective behaviors (i.e., vaccination). At the same time, since many individuals still refuse vaccination in spite of the accumulating evidence of the major health risks of COVID-19, fear appears to have a limited effect on vaccination intentions. Its influence may be limited, among others, by conspiracist beliefs about COVID-19, which undermine, within the PMT framework, the second pillar (i.e., the coping appraisal) of the motivation towards protecting oneself through vaccination among people who hold such beliefs. Beliefs in COVID-19 conspiracy theories have been highlighted as an important motivator of people’s vaccine hesitancy (Romer and Jamieson, 2020; Loomba et al., 2021). Many conspiracy theories and misinformation that have circulated during the current pandemic undermine the importance, safety or effectiveness of COVID-19 vaccines, building on and extending pre-pandemic conspiracist ideas about vaccines in general (e.g., Burki, 2019). Together with the other facets of conspiracist beliefs about the new coronavirus, which target the “true” nature of the disease or its origin and consequently seed doubt over the trustworthiness of state and medical authorities, these ideas negatively affect vaccine intentions (Freeman et al., 2020; Milošević Đorđević et al., 2021). In the PMT framework, this influence can be explained through the fact that such beliefs undermine people’s confidence in the efficacy of vaccination as a response to the threat posed by the new coronavirus. Moreover, they may also induce false appraisals concerning some high personal health costs that would be incurred by those who uptake the COVID-19 vaccine. Both these effects lead people who hold conspiracist beliefs on this topic to appraise vaccination as having a low coping potential in relation to the COVID-19 threat, even though they may simultaneously perceive this disease as highly threatening. Past research highlighted mixed relations between beliefs in such conspiracy theories and COVID-19 related perceived risk and fear: while some investigations found a positive relationship between them (e.g., Sallam et al., 2020), other found the opposite (Romer and Jamieson, 2020). Our main focus in on the joint effect that conspiracist beliefs and COVID-19 fear may have on people’s intention to uptake the available vaccines against the new coronavirus. In this respect, people who adhere to conspiracy theories on this topic might undervalue vaccines as a coping response to this disease, by considering them as a useless and/or dangerous medical intervention. This effect could be independent from the influence of fear and of the associated threat appraisal, in that people who feel intense anxiety concerning COVID-19 while also holding conspiracist beliefs might thus refuse the vaccine as a mean to protect themselves from the risks they fear, which ultimately implies the cancellation of the functional and protective character of this health-related emotion.

The present study also considers other factors that were previously highlighted as associated to people’s intentions towards vaccination, in order to investigate their influences in the specific population of our research and to control them in the examination of the joint effect of fear of the new coronavirus and conspiracist beliefs. Among these factors, we address perceived risk of catching COVID-19, which is another facet of risk perception concerning the coronavirus besides the one focused on its health effects, and which has been found to be positively related to vaccine acceptance (Salali and Uysal, 2020). We also consider trust in medical experts, as past studies showed that lack of trust in biomedical research and medical authorities is a significant reason for vaccine hesitancy during the current pandemic (Palamenghi et al., 2020; Latkin et al., 2021; Troiano and Nardi, 2021). Lower levels of education were also found to be associated to lower intentions to uptake the COVID-19 vaccines (e.g., Ruiz and Bell, 2021), while the results of previous studies on the relationships between these intentions and gender were mixed (e.g., Latkin et al., 2021; Paul et al., 2021; Zintel et al., 2022).

Another factor that past research highlighted as significant for COVID-19 vaccine acceptance was individual’s attitude toward vaccines in general (Allington et al., 2021; Troiano and Nardi, 2021). In addition to the overall evaluation of vaccines that the concept of attitude entails, we also target a related dimension, i.e., felt ambivalence towards vaccination. Ambivalence is generated by exposure to opposite and diverse arguments on a topic (Priester and Petty, 2001). This is also the case during the current pandemic, as people are exposed to multiple and conflictive information and positions about the virus and the newly-developed vaccines. As public health experts stated, there is an “infodemic” of misinformation on these topics that promote messages opposite to those from biomedical science and authorities (Zarocostas, 2020). One of the effects of exposure to this wide array of positions may be that of inducing ambivalence towards COVID-19 vaccines in many people, which entails that they would simultaneously hold positive and negative evaluations of this target, as suggested by past research on the influence of competing mass-media information on ambivalence (Mutz, 2006). In the health context, Kim et al. (2019) found that exposure to competing information that highlights both benefits and side effects of vaccination induces ambivalence and leads to lower intentions to receive the vaccine against seasonal influenza virus, in line with previous studies showing that ambivalence induces hesitancy to engage in relevant behaviors (Hänze, 2001). This suggests that ambivalence toward vaccines could be another negative factor of the intentions to vaccinate against COVID-19.

The main aim of the current study is to examine the effects of fear of COVID-19 and conspiracist beliefs about the new coronavirus on vaccination intentions. Previous studies have investigated and documented only the independent effect of each of these factors, but whether the effects of fear on vaccination intentions depends on people’s conspiracist beliefs about COVID-19 remains an open question. We aim to address this research gap examining the interaction between these factors, specifically the moderating effect of conspiracist beliefs on the relationship between fear and vaccination intentions. Our main assumption is that conspiracist beliefs not only affect vaccination intentions, but that they also limit the positive influence of COVID-19 fear on these intentions. Specifically, we expect that fear would be less influential in increasing intentions to uptake the COVID-19 vaccine in people who hold stronger conspiracist beliefs. Moreover, we also aim to study the effects of other factors highlighted by past research as associated to people’s vaccination intentions. Besides perceived risk of catching COVID-19, trust in medical experts, and attitude towards vaccination, we also investigate the potential role of ambivalence towards vaccines, highly relevant in the current “infodemic” surrounding this topic, as another deterrent of the intentions to vaccinate against COVID-19. In this regard, we aim to further explore the routes of influence of conspiracist beliefs on vaccination intention by examining ambivalence towards vaccines as a potential mediator of this effect. We expect people who hold stronger conspiracist beliefs about COVID-19 to be also more ambivalent about vaccination, and this to further render them less prone to uptake the COVID-19 vaccine. Finally, we also examine the effects of three socio-demographic variables, i.e., gender, age and education, which have been frequently considered by past research on differences in COVID-19 related fear (Lippold et al., 2020; Luo et al., 2021; Wang et al., 2022).

## 2. Method

The present study used previously validated scales to measure the constructs addressed. The data was collected on a convenience sample from the Romanian population. A quantitative approach was then used on the data in order to examine the relationships between study variables.

### 2.1. Research procedure

We conducted a web-based cross-sectional survey in October 2021, when Romania’s full vaccination rate was 37%, less than half the EU average of 75% (ECDC, 2021). The survey was distributed via social media platforms (i.e., Facebook) to students from two Universities in Romania with the invitation to fill it and to also distributed towards other potential participants from their acquaintances. Facebook is the social media platform with the widest use among all socio-demographic groups of the Romanian public. The inclusion criteria were: age over 18 years, residence in Romania and not having taken a COVID-19 vaccine by the time of the participation in the study. The research design and methods followed the ethical guidelines of the 2013 Helsinki Declaration. Participation in the study was voluntary, participants were given the opportunity to withdraw at any time, they were ensured about the confidentiality and anonymity of their answers, and they gave their informed consent by choosing the consent statement (i.e., “I understood the study’s aim, my rights, and I agree to participate in this study”). All participants ticked this response option. No other personal information about participants was collected besides the socio-demographics included in the survey, and all data was used solely for research purposes. The time needed to answer the survey questions was approximatively 20 min.

### 2.2. Participants

Five hundred eighty-two participants completed the online survey. Eighteen answered “Yes” to the item about having taken a COVID-19 vaccine, consequently their answers were excluded from the database. The final sample includes 564 participants, aged 18–61 years (*M* = 25.01, SD = 7.79). Most respondents (i.e., 348) were females, and 362 had a university degree (see Table 1).

**Table 1:** Sample demographics (*N* = 564).

	*N*	%
*Age group*		
18–25	453	80.3
> 25	111	19.7
*Gender*		
Male	216	38.3
Female	348	61.7
*Education*		
High-school	202	35.8
University	362	64.2

### 2.3. Measures

*Intention to take the COVID-19 vaccine* was measured using a single item with a yes/no response scale (e.g., “Would you vaccinate against COVID-19 with one of the vaccines developed so far?”).

The *Fear of COVID-19* scale by Ahorsu et al. (2020) was used to assess participants’ fear of the coronavirus. Participants rated each of the 7-item using a response scale ranging from 1 to 5, where 1—*strongly disagree* and 5—*strongly agree* (e.g., “I am most afraid of coronavirus-19”). The scale showed good reliability coefficients (Alpha = 0.85; McDonald’s *ω* = 0.86). An average score was computed for each participant, with higher scores indicating the severity of the fear of COVID-19.

*Beliefs in COVID-19 conspiracy theories* were measured using the 10-item scale by Biddlestone et al. (2020). Participants rated their agreement with each of the ten items using a scale ranging from 1 to 7, where 1—*strongly disagree* and 7—*strongly agree* (e.g., “The implementation of 5G technology is a means of deliberately spreading Coronavirus”). The scale showed good reliability coefficients (Cronbach’s Alpha = 0.92; McDonald’s *ω* = 0.92). The average score for each participant was computed, with higher scores suggesting a higher tendency to believe in conspiracy theories.

*Perceived risk of catching COVID-19* was assessed using one item scale adapted from Kerr et al. (2021) (i.e., “How likely do you think it is that you will be directly and personally affected by catching the coronavirus/COVID-19 in the next 6 months?”) using a 7-point response scale (i.e., 1—*not at all likely*, 7—*very likely*).

Participants reported their *trust in medical experts* using the two items adapted from Kerr et al. (2021) using a 7-point scale (i.e., 1—*cannot be trusted at all*, 7—*can be trusted a lot*). An average score was computed for each participant, with higher levels indicating trust in medical experts.

*Attitude toward vaccines* was measured using two items assessing individuals’ beliefs that vaccines represent a safe and reliable means to help avert the spread of preventable diseases and that they represent one of the most significant contributions to public health using a 5-point scale, ranging from 1—strongly disagree to 5—strongly agree (Kerr et al., 2021). The average score for each participant was computed, with higher scores suggesting a more positive attitude toward vaccination.

*Ambivalence towards vaccination* was assessed using a six items scale adapted from Lipkus et al. (2001) as formerly used by Kim et al. (2019). Participants rated their agreement using a 7-point scale ranging from 1—*strongly disagree* to 7—*strongly agree*. The statements presented conflicting thoughts and feelings towards vaccinations (e.g., “I have strong feelings both for and against flu vaccination”). Each participant obtained an average score, with higher scores suggesting a higher ambivalence toward vaccination. The scale showed good reliability coefficients (Alpha = 0.90; McDonald’s *ω* = 0.91).

The accuracy of the Romanian translation of the scales was checked through the back-translation method. We also assessed participants’ gender, age, and education level (i.e., high school or bachelor’s degree).

## 3. Results

### 3.1. Relationships between variables

The associations between study variables are presented in Table 2, together with their descriptive statistics. More than half of participants (i.e., 52.8%) declared that they have no intention to take a COVID-19 vaccine. Results indicated positive relationships between the intention to take the COVID-19 vaccine and attitude towards vaccines, trust in medical experts, fear of COVID-19 and perceived risk of catching the new coronavirus, while beliefs in COVID-19 conspiracy theories and ambivalence towards vaccination emerged as negatively related to these intentions. Moreover, beliefs in COVID-19 conspiracy theories and ambivalence towards vaccination were positively associated to fear of COVID-19, and negatively related to attitude towards vaccines and trust in medical experts. Fear of COVID-19 was also positively associated perceived risk of catching COVID-19 and attitude towards vaccines. Trust in medical experts emerged as strongly correlated to attitude towards vaccines, and both were positively associated to perceived risk of catching the new coronavirus. Although none of the demographic variables were associated to the intention to take the COVID-19 vaccine, results suggest that females reported more intense fear and a higher perceived risk of catching COVID-19, but also more ambivalence towards vaccination, while older participants held stronger beliefs in COVID-19 conspiracy theories.

**Table 2:** Descriptive statistics and associations between study variables.

	Descriptive statistics		1	2	3	4	5	6	7	8	9
1. Fear of COVID-19	1.88	0.73									
2. Beliefs in COVID-19 conspiracy theories	1.97	0.87	0.10^*^								
3. Ambivalence towards vaccination	3.51	1.62	0.20^**^	0.40^**^							
4. Perceived risk of catching COVID-19	3.78	1.57	0.22^**^	−0.05	0.12^**^						
5. Attitude toward vaccines	3.82	1.12	0.10^*^	−0.48^**^	−0.17^**^	0.17^**^					
6. Trust in medical experts	5.11	1.26	0.05	−0.42^**^	−0.19^**^	0.14^**^	0.56^**^				
7. Gender	61.7% females		0.15^**^	0.06	0.18^**^	0.10^*^	0.02	−0.05			
8. Age	24.67	7.42	0.03	0.11^*^	0.06	0.05	0.01	0.04	0.06		
9. Education	64.2% bachelor		0.03	−0.03	0.01	0.05	0.07	0.03	0.23^**^	0.14^**^	
10. Intention to take the COVID-19 vaccine	47.2% Yes		0.10^*^	−0.40^**^	−0.30^**^	0.15^**^	0.48^**^	0.35^**^	0.05	−0.02	0.05

The measure of association between the quantitative variables (i.e., mean responses on the six scales and age) was Pearson correlation. The measure of association between the quantitative and the categorical variables (i.e., gender, education and vaccination intention) was the point-biserial correlation. The measure of association between the categorical variables was Cramér’s V. ^*^p < 0.05; ^**^p < 0.01

### 3.2. Predictors of the intention to take the COVID-19 vaccine

Next, we used multiple binary logistic regression analysis to examine the relationships between our presumed set of factors, i.e., fear of COVID-19, beliefs in COVID-19 conspiracy theories, ambivalence towards vaccination, perceived risk of catching COVID-19, attitude towards vaccines, trust in medical experts, introduced as predictors, and the intention to take the COVID-19 vaccine. In order to test the hypothesized interaction between fear of COVID-19 and beliefs in COVID-19 conspiracy theories, we also introduced this interaction term as a predictor in the regression model. The effects of age, gender, and education on these intentions were also checked and controlled by using them as separate predictors. All quantitative predictors (i.e., overall scores on each scale and age) were mean-centered, by subtracting the mean from each individual score, in line with multiple regression guidelines (Aiken and West, 1991).

The regression model was statistically significant, *χ*^2^(10) = 239.94, *p* < 0.001, Nagelkerke *R*^2^ = 0.46. Results, presented in Table 3, indicated significant relationships between the intention to take the COVID-19 vaccine and fear of COVID-19, beliefs in COVID-19 conspiracy theories, ambivalence towards vaccination, perceived risk of catching COVID-19, attitude towards vaccines, gender and education. Participants with higher levels of fear, those with weaker beliefs in COVID-19 conspiracy theories, those less ambivalent towards vaccination, those perceiving a higher risk of catching the new coronavirus, those with more positive attitudes towards vaccines, females and those with a bachelor’s degree had higher odds of intending to take the COVID-19 vaccine than their respective counterparts.

**Table 3:** Summary of logistic regression analysis for variables predicting intention to take the COVID-19 vaccine.

Variable	B (SE)	Wald’s *χ*^2^	*p*	Odds ratio	95% CI for the odds ratio
Fear of COVID-19	0.54 (0.18)	9.36	0.002	1.71	1.21–2.41
Beliefs in COVID-19 conspiracy theories	−0.52 (0.17)	9.92	0.002	0.60	0.43–0.82
Ambivalence towards vaccination	−0.38 (0.08)	22.43	< 0.001	0.69	0.59–0.80
Perceived risk of catching COVID-19	0.15 (0.07)	3.95	0.047	1.16	1.002–1.34
Attitude toward vaccines	0.89 (0.13)	44.88	< 0.001	2.44	1.88–3.17
Trust in medical experts	0.11 (0.11)	0.94	0.333	1.11	0.90–1.38
Fear of COVID-19 × Beliefs in COVID-19 conspiracy theories	−0.92 (0.22)	17.37	< 0.001	0.40	0.26–0.61
Gender (reference: female)	−0.48 (0.23)	4.22	0.04	0.62	0.39–0.98
Age	0.01 (0.02)	0.32	0.578	0.99	0.96–1.02
Education (reference: bachelor’s degree)	−0.69 (0.23)	8.89	0.003	0.50	0.32–0.79

CI = confidence interval; Reference category for dependent variable (intention to take the COVID-19 vaccine) is “No.”

The interaction between fear of COVID-19 and beliefs in COVID-19 conspiracy theories also emerged as a significant predictor. We explored this interaction by analyzing the effects of fear of COVID-19 on the intention to take the COVID-19 vaccine separately in participants who hold strong COVID-19 conspiracist beliefs and in those with weaker beliefs in such conspiracy theories. To this end, we split the sample according to the median of the distribution of scores on the measure of beliefs in COVID-19 conspiracy theories (Mdn = 1.70). Then we conducted separate binary regression analyses on each of these two groups defined by their strength of beliefs in COVID-19 conspiracy theories (low vs. high), examining the relationships between the intention to take the COVID-19 vaccine and the other predictors (i.e., fear of COVID-19, ambivalence towards vaccination, perceived risk of catching COVID-19, attitude towards vaccines, trust in medical experts, gender, age and education). The main results of these separate analyses (i.e., the odds ratios of each predictor and their 95% confidence intervals) are presented in Table 4. They indicated that in the group of participants with weaker beliefs in COVID-19 conspiracy theories the pattern of relationships between the intention to take the vaccine and the set of predictors is similar to the one that emerged in the previous analysis on the whole sample. Fear of COVID-19, perceived risk of catching COVID-19 and attitude towards vaccines were found to be associated to higher odds of taking the COVID-19 vaccine, while ambivalence towards vaccination, the male gender and having only high school education to vaccine refusal. On the other hand, the results of the analysis on participants with stronger beliefs in COVID-19 conspiracy theories suggested that only attitude towards vaccines and ambivalence towards vaccination are significant predictors of intentions to take the COVID-19 vaccine in this group. Critically, and in line with our hypothesis, fear of COVID-19 emerged as not related to vaccination intentions in these participants who hold stronger conspiracist beliefs.

**Table 4:** Summary of logistic regression analysis for variables predicting intention to take the COVID-19 vaccine in the two groups defined by strength of beliefs in COVID-19 conspiracy theories.

	Strength of beliefs in COVID-19 conspiracy theories			
	Low (*N* = 270)		High (*N* = 276)	
	Odds ratio	95% CI for the odds ratio	Odds ratio	95% CI for the odds ratio
Fear of COVID-19	4.83^**^	2.54–9.18	0.78	0.52–1.18
Ambivalence towards vaccination	0.64^**^	0.51–0.80	0.70^**^	0.56–0.88
Perceived risk of catching COVID-19	1.32^*^	1.05–1.67	1.06	0.87–1.29
Attitude toward vaccines	2.61^**^	1.66–4.10	2.63^**^	1.85–3.75
Trust in medical experts	1.17	1.05–1.67	1.09	0.83–1.43
Gender (reference: female)	0.44^*^	0.22–0.88	0.71	0.37–1.37
Age	0.99	0.94–1.05	0.99	0.95–1.03
Education (reference: bachelor’s degree)	0.46^*^	0.23–0.93	0.55	0.29–1.03
Model summary				
*χ* ^2^	101.54^**^		58.98^**^	
*df*	8		8	
Nagelkerke *R*^2^	0.44		0.28	

CI = confidence interval; Reference category for dependent variable (intention to take the COVID-19 vaccine) is “No”. ^**^p < 0.01; ^*^p < 0.05

### 3.3. Differences in fear of COVID-19 between participants with stronger and those with weaker conspiracist beliefs

Finally, we compared the two groups defined by their strength of beliefs in COVID-19 conspiracy theories on fear of COVID-19, to check whether the different pattern of relationships between fear and vaccination intentions in these groups is associated to differences in the intensity of fear of COVID-19 that they experience. The results of the *t*-test showed no significant differences between groups, *t*(533) = 0.97; *p* = 0.33, suggesting that participants with weaker beliefs in COVID-19 conspiracy theories experienced similar levels of fear of COVID-19 (*M* = 1.86, SD = 0.67) to those experienced by participants holding stronger conspiracist beliefs (*M* = 1.92, SD = 0.79). Therefore, the null effect of this fear on vaccination intentions in this latter group cannot be explained by them being less fearful of COVID-19 than participants with weaker conspiracist beliefs.

### 3.4. Ambivalence towards vaccination as mediator of the effect of COVID-19 conspiracist beliefs on vaccination intention

**Figure 1:**
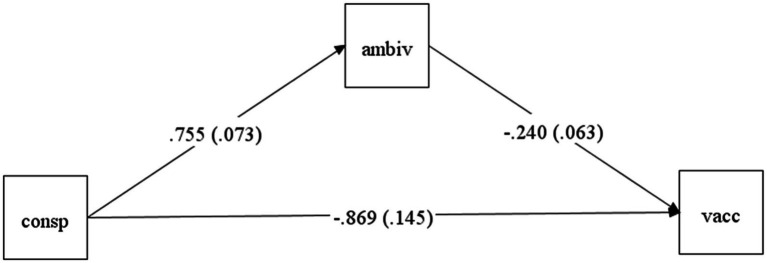
The mediation path from conspiracist beliefs to vaccination intention through vaccine ambivalence. consp = conspiracist beliefs; ambiv = vaccine ambivalence; vacc = vaccination intention. The estimates represent unstandardized effects with SE values in parenthesis and are significant (*p* < 0.001).

As vaccination intention is binary measured, a counterfactually defined causal mediation method was used in MPlus version 8.8 (Muthen et al., 2016) to test the mediation effect of vaccine ambivalence in the relationship between COVID-19 conspiracist beliefs and vaccination intention. The mediation approach decomposes the total effect into (a) the *natural indirect effect*, which represents the effects of the independent variable on the outcome through the mediator while blocking the direct effect; and (b) the *pure natural direct effect*, which represents the direct effect of the variable on the outcome while blocking the effect through the mediator (Vanderweele, 2015; Nguyen et al., 2020; Rijnhart et al., 2021). Furthermore, maximum likelihood was used to estimate the regression parameters, and 10,000 samples were drawn for bootstrapping (Valente et al., 2020). No missing data was observed. The results of this analysis are presented in Table 5 and Figure 1.

**Table 5:** Bootstrap confidence intervals using logit regression for vaccination intention.

Confidence intervals of total, indirect, and direct effects based on counterfactuals					
	Lower 2.5%	Lower 5%	Estimate	Upper 5%	Upper 2.5%
*Effects from conspiracist beliefs to vaccination intention*					
Tot natural IE	−0.056	−0.052	−0.036	−0.021	−0.018
Pure natural DE	−0.157	−0.152	−0.132	−0.108	−0.103
Total effect	−0.188	−0.184	−0.168	−0.149	−0.146
*Odds ratio for vaccine intention*					
Tot natural IE	0.757	0.769	0.838	0.903	0.916
Pure natural DE	0.315	0.331	0.427	0.535	0.559
Total effect	0.265	0.279	0.357	0.443	0.461

Tot natural IE = total natural indirect effect; Pure natural DE = pure natural direct effect.

Results suggested that COVID-19 conspiracist beliefs significantly predicted vaccination intention (*b* = −0.86, *p* < 0.001), suggesting that individuals with stronger conspiracist beliefs tend to refuse vaccination. Secondly, conspiracist beliefs significantly predicted vaccine ambivalence (*b* = 0.75, *p* < 0.001), and ambivalence significantly and negatively predicted vaccine intention (*b* = −0.24, *p* < 0.001), suggesting that individuals with higher levels of ambivalence towards vaccination tend to refuse the COVID-19 vaccination (see Figure 1).

The total natural indirect effect, in probability metric, is estimated at −0.03 and is significant (95% CI: −0.056, −0.018), suggesting the effect of conspiracist beliefs on vaccine intention through ambivalence, while blocking the direct intervention effect, is significant. The direct effect (pure natural DE) in probability metric is estimated as −0.13 (95% CI: −0.157, −0.103). The total effect in probability metric of −0.168 is significant. The direct effect of conspiracist beliefs on vaccine intention, while blocking the effect of ambivalence, is also significant (95% CI: −0.188, − 0.146). The odds ratio (OR) in this case characterized a common outcome (i.e., > 10% of the time); thus, they do not accurately measure relative risk and do not have causal interpretation, but the estimates can still be used to test the presence of natural effects (Vanderweele and Vansteelandt, 2010; Valeri and Vanderweele, 2013; Muthen et al., 2016; Feingold et al., 2019; Rijnhart et al., 2021).

## 4. Discussion

During the current pandemic, it is critical to uncover the determinants of people’s hesitancy towards the available COVID-19 vaccines. Although the disease provoked by the new coronavirus is potentially health damaging and even deadly, our findings indicate that the fear generated by these risks fosters the intention to vaccinate only in people who have low adherence to conspiracy theories. The positive influence of fear of COVID-19, associated with the high risk of contracting this disease and to its severity, on vaccination intentions and attitudes has previously found when this factor was analyzed independently (e.g., Bendau et al., 2021; Chu and Liu, 2021; Lielsvagere-Endele et al., 2022; Nguyen et al., 2022; Rosli et al., 2022). However, our study suggests that conspiracist beliefs on this topic moderate this effect by limiting and even canceling it. Specifically, we found that the intentions to uptake the COVID-19 vaccines are not influenced by fear in people who hold stronger conspiracist beliefs. This interference in the relationships between fear of the perceived risks of COVID-19 and vaccination intentions adds to the direct negative effect of conspiracist beliefs on these intentions, which also emerged in our results, in line with past studies (e.g., Loomba et al., 2021; Milošević Đorđević et al., 2021).

Contrarily to past results on the relationships between conspiracist thinking about COVID-19 and fear of this disease (e.g., Romer and Jamieson, 2020; Sallam et al., 2020), our findings indicate that the participants in our sample who believe in conspiracy theories about COVID-19 were equally fearful of the risks associated to this disease as those who do not endorse such beliefs. This suggests that the tendency to refuse the COVID-19 vaccine of people who hold conspiracist beliefs is not a consequence of their eventual underestimation of the risks that this disease entails and of their feelings of security on this issue. Their vaccine hesitancy is, instead, motivated by their misperception of the newly developed COVID-19 vaccines within the lines promoted by the conspiracy theories, which aim to raise doubts concerning their safety, necessity and efficacy (Freeman et al., 2020; Loomba et al., 2021). Consequently, they do not consider vaccines as a solution to the COVID-19 threat, although they acknowledge and emotionally respond to the risks that it involves. In the PMT framework, this pattern of findings indicates that individuals who hold COVID-19 conspiracist beliefs are less motivated to adopt the recommended protective behavior (i.e., vaccination) against this disease because of their low coping appraisal in relation to this behavior, in spite of their high threat appraisal.

The finding that fear does not lead to stronger intentions to uptake the COVID-19 vaccine in people who hold conspiracist beliefs also suggests that mass communication strategies aiming to reduce vaccine hesitancy through fear appeals, which highlight the damaging effects of the disease, may have a limited impact in this population. The use of public campaigns highlighting the risks of the COVID-19 disease has been recommended as a strategy to increase vaccine acceptance (Chu and Liu, 2021; Motta et al., 2021). Nevertheless, our findings suggest that this type of message emphasizing risk and instilling fear may indeed foster vaccination intentions only in people whose perspectives on the current pandemic are not contaminated by conspiracy theories. Individuals who hold conspiracist beliefs, on the other hand, may be less persuaded by such fear-based appeals, because of their reluctance to accept vaccination as an adaptive coping response. Their pre-existent conspiracist perspectives on vaccination and COVID-19 function as cognitive filters that render them distrustful of the benefits of COVID-19 vaccines and/or suspicious of the consequent personal costs that would be incurred if they resort to this intervention. Consequently, such public campaigns would only increase the intensity of fear in people who hold conspiracist beliefs, but without reaching their actual objective of raising vaccine acceptance. Generally, previous studies suggested that communication efforts that aim to increase vaccine confidence should be diverse and tailored to different categories of public, differentiated according to their emotional reactions and prior beliefs about the new coronavirus and about the vaccines (Chou and Budenz, 2020; Su et al., 2020). Critically, our findings further emphasize the importance of combating the disinformation spread by COVID-19 conspiracy theories in order to improve vaccination intentions, in line with past studies (Romer and Jamieson, 2020; Motta et al., 2021; Pertwee et al., 2022).

Our results also parallel previous findings regarding other significant factors of COVID-19 vaccination intentions. Specifically, we found that the intentions to uptake the COVID-19 vaccine are stronger in people who perceive a higher risk of being contaminated by the new coronavirus, in those who have higher levels of trust in medical experts and more positive attitudes towards vaccines in general, in line with past results on these relationships (Palamenghi et al., 2020; Salali and Uysal, 2020; Allington et al., 2021). Similar to past findings, the college-educated participants in our sample had higher intentions to vaccinate (Milošević Đorđević et al., 2021; Ruiz and Bell, 2021). Males in our sample were more vaccine hesitant than women, a result that differs from those of the gender comparisons across studies recently reviewed by Zintel et al. (2022).

Moreover, ambivalence emerged in our results as a novel factor within the set of determinants that have been identified by extant research on vaccination intentions. Specifically, people who feel ambivalent about vaccination are more hesitant to uptake the COVID-19 vaccine, paralleling previous results on the importance of ambivalence for other types of vaccines (Kim et al., 2019). But this factor may be even more significant during the current public health crisis, when there is a lot of misinformation propagated in the media contradicting the health experts’ positions on the topic of the new coronavirus and of the vaccines that have been developed against it. This “infodemic” is likely to generate ambivalence towards the COVID-19 vaccines in many people, which should be considered in the development of public communication messages that encourage vaccination. The results of our mediation analysis support this assumption, as vaccine ambivalence emerged as a mediator of the effects of conspiracist beliefs about COVID-19 on vaccination intention. This also highlights a particular route of influence through which conspiracist beliefs foster vaccine hesitancy, that of generating ambivalence towards vaccines. They entail negative evaluations of their importance, safety or effectiveness, which contradict the official public health messages and thus induce ambivalent appraisals in people who hold such beliefs. This contradictory nature of ambivalent appraisals and the associated uncertainty regarding the “true” effects of the vaccine further renders people reticent towards vaccination, in line with empirical results from other research areas, for instance those that highlighted uncertainty as a mediator of the negative effects of conspiracist beliefs on intentions to engage in climate change mitigation behaviors (Jolley and Douglas, 2014).

The main strength of our study is the in-depth exploration of the effects of conspiracist beliefs about and fear of COVID-19 on vaccination intention, by examining not only their independent influences but also their interaction, as well as vaccine ambivalence as mediator of the effect of conspiracist beliefs. One of the limits of our research is the use of a dichotomic measure of vaccination intention instead of a more fine-grained measure that would have capture the variability in the strength of these intentions. Moreover, the present study relies on self-report measures, uses a cross-sectional design that cannot determine causal relationships, data was collected through an online survey that may generate sampling bias, and most participants were young (i.e., under 25 years old) and university educated, all these aspects limiting the generalizability of its findings. It is also important to note that our research was conducted in Romania, on a population with a low COVID-19 vaccination percentage so far in comparison to the other EU countries, and its findings indicate that a high proportion of our sample reject the COVID-19 vaccine, in line with past results on the same population (Maftei and Holman, 2021). These relationships should be also examined in countries with higher COVID-19 vaccination rates, by taking into account socio-cultural factors that may further explain people’s vaccine hesitancy, such as trust in information from government sources or confidence in the health system (Lazarus et al., 2021; Al-Amer et al., 2022). Further studies should also examine the relationships between fear of COVID-19 and conspiracist beliefs in a more granular manner, by differentiating between different types of fears concerning the current pandemic (e.g., fear of illness itself vs. that of the social or economic consequences, in line with Bendau et al., 2021) and between people holding beliefs in different conspiracist ideas (e.g., “hoax”-related theories vs. those about the virus being manufactured in a laboratory, see Imhoff and Lamberty, 2020).

In conclusion, this study suggests that the emotional reactions induced by the perceptions of the risks of COVID-19, i.e., fear, positively influences the intention to be vaccinated against the new coronavirus, but only in people who do not endorse conspiracist ideas on this topic. This cancelling effect of conspiracist beliefs on the relationship between fear and vaccination intentions highlights the need to complement health communication messages focused on emphasizing the risks of COVID-19 with strategies to combat disinformation.

## Data availability statement

The raw data supporting the conclusions of this article will be made available by the authors, without undue reservation.

## Ethics statement

The studies involving human participants were reviewed and approved by the Faculty of Psychology and Education Sciences, Alexandru Ioan Cuza University of Iaşi. The patients/participants provided their written informed consent to participate in this study.

## Author contributions

All authors listed have made a substantial, direct, and intellectual contribution to the work and approved it for publication.

## Conflict of interest

The authors declare that the research was conducted in the absence of any commercial or financial relationships that could be construed as a potential conflict of interest.

## Publisher’s note

All claims expressed in this article are solely those of the authors and do not necessarily represent those of their affiliated organizations, or those of the publisher, the editors and the reviewers. Any product that may be evaluated in this article, or claim that may be made by its manufacturer, is not guaranteed or endorsed by the publisher.
